# Non-native megaherbivores: the case for novel function to manage plant invasions on islands

**DOI:** 10.1093/aobpla/plv085

**Published:** 2015-07-18

**Authors:** Dennis M. Hansen

**Affiliations:** Institute of Evolutionary Biology and Environmental Studies, University of Zurich, Winterthurerstrasse 190, CH-8057 Zurich, Switzerland

**Keywords:** Control, ecosystem function, eradication, giant tortoises, herbivory, invasive plants, restoration

## Abstract

There is a heated debate about whether all non-native species are ‘guilty until proven innocent’, or whether some should be accepted or even welcomed. Further fanning the flames, I here present a case where introductions of carefully vetted, non-native species could provide a net conservation benefit. On many islands, native megaherbivores (flightless birds, tortoises) recently went extinct. Here, rewilding with carefully selected non-native species as ecological replacements is increasingly considered a solution, reinstating a herbivory regime that largely benefits the native flora. Based on these efforts, I suggest that restoration practitioners working on islands without a history of native megaherbivores that are threatened by invasive plants should consider introducing a non-native island megaherbivore, and that large and giant tortoises are ideal candidates. Such tortoises would be equally useful on islands where eradication of invasive mammals has led to increased problems with invasive plants, or on islands that never had introduced mammalian herbivores, but where invasive plants are a problem. My proposal may seem radical, but the reversibility of using giant tortoises means that nothing is lost from trying, and that indeed much is to be gained. As an easily regulated adaptive management tool, it represents an innovative, hypothesis-driven ‘innocent until proven guilty’ approach.

## Abstract (precis)

I propose that in specific ecosystems, introductions of carefully vetted, non-native species could provide a net conservation benefit. On many islands, non-native species are increasingly used as ecological replacements for extinct native species, reinstating a herbivory regime that largely benefits the native flora. Based on these efforts, I suggest introductions of non-native megaherbivores to islands that are threatened by invasive plants, but which never harboured native megaherbivores, as a conservation management tool. I argue that large and giant tortoises are ideal candidates. As an easily-regulated adaptive management tool, my proposal represents an innovative, hypothesis-driven approach to island conservation.

## Introduction

There is a heated debate as to whether to accept, or even welcome, a long-term presence of some non-native species in ecosystems (Davis *et al*. 2011), or whether to apply a ‘guilty until proven innocent’ approach to all of them (Simberloff *et al*. 2013). However, non-native species are increasingly being recognized for their potential usefulness in conservation and restoration (Ewel and Putz 2004; Goodenough 2010; Schlaepfer *et al*. 2011). Likely further fanning the flames, I here suggest an innovative approach for controlling invasive plants on certain islands by adding a novel ecological function without a historical analogue. I would like to acknowledge up front that the critical nature of conservation and restoration challenges on islands puts an obvious premium on precaution and the need for case-specific empirical evidence. There is no doubt that non-native species can be extremely harmful, especially on oceanic islands, where many native and endemic species have been rapidly driven to extinction by introduced predators (Savidge 1987; Nogales *et al*. 2004) or pathogens (Warner 1968; Wyatt *et al*. 2008). Other major problems are caused by invasive plants and mammalian herbivores, who together rank as two of the main threats to native island plant biodiversity (Caujapé-Castells *et al*. 2010).

While islands are disproportionately impacted by invasive species, in turn, many pioneering management methods such as eradications were developed and successfully deployed on islands (Veitch and Clout 2002). For example, the increasingly effective eradications of invasive mammalian herbivores have led to striking recoveries of native vegetation in many islands (e.g. Hamann 1993; Aguirre-Muñoz *et al*. 2011; Alves *et al*. 2011; Beltran *et al*. 2014). Ironically, herbivore eradications can also lead to a worsening of other problems; e.g. causing islands to be smothered by rapid-growing invasive plants formerly held in check by the herbivores (Zavaleta *et al*. 2001). In some cases, these explosions of invasive plant growth can be relatively short-lived. For example, on Sarigan Island, Mariana Islands, the introduced vine *Operculina turpethum* var. *ventricosa* (Bertero) Staples & D.F. Austin rapidly suffocated the island after the eradication of pigs and goats (Kessler 2002). This vine subsequently lost steam within a decade, allowing one native tree species, but also at least two other invasive vine species, to gain ground (Kessler 2011). In other cases, after eradications of introduced herbivores, problems with invasive plants increase and remain worse than before. For example on Round Island, Mauritius, where eradications of invasive goats and rabbits between 1978 and 1986 led to a massive increase in invasive plant populations (Bullock *et al*. 2002). These problems continued unabated until a recent conservation introduction of hundreds of giant tortoises to Round Island in an attempt to restore a native herbivory regime (Griffiths *et al*. 2013*a*); see the section on lessons from tortoise rewilding, below, for details.

Current invasive plant control measures adopt a suite of tactics (biological control agents, chemicals and mechanical removal techniques) intended to attack the unrestrained growth of one or a few species. Among these methods, biological control is regarded as a viable long-term solution (Clewley *et al*. 2012; Seastedt 2015). Typically, a specialist natural enemy from the native range of the invasive plant is introduced with the aim of controlling the invasive plant. These control agents are invariably invertebrate herbivores or pathogens, chosen for their host or feeding specificity. Despite a relatively successful history of applications, these agents may become difficult to control long-term. Critically, given the size and life histories of the control agents, such introductions are irreversible. Even with some justified optimism in relation to the future use of biological control agents (Seastedt 2015), using only species-specific control agents is unrealistic given that the number of invasive plants on islands is forecast to increase (Sax and Gaines 2008). There thus remains an unprecedented and growing need for innovative solutions and tools to control, eradicate or mitigate the impacts of invasive plants (Lambertini *et al*. 2011), especially on oceanic islands.

I will argue in the following that, on some islands, the establishment of a novel ecological function might be one way forward as a solution to this challenge. Specifically, I am focussing on introducing carefully vetted, non-native megaherbivores to islands without a history of native megaherbivores that are threatened by invasive plants. My proposal is inspired by encouraging first results emerging from island rewilding projects, where extant non-native herbivores are being introduced to functionally replace recently extinct native herbivores. Based on these, I believe that large and giant tortoises are ideal candidates for introducing a novel herbivory regime. I posit that such a herbivory regime would largely benefit native plants, by shifting the competitive advantage away from invasive plants and towards native and endemic ones. Tortoise megaherbivores would be equally useful on islands where eradication of invasive mammals has led to increased problems with invasive plants, or on islands that never had introduced mammalian herbivores, but where invasive plants are a problem. A useful reference framework for when and how to undertake such introductions can be provided by the recently revised and expanded Guidelines for Reintroductions and Other Conservation Translocations (IUCN/SSC 2013). My proposal can be viewed as conservation translocations that are kindred spirits of rewilding projects, but which rely only on functional ecological arguments without referring to explicit historical baselines.

In continental ecosystems, native as well as non-native, large-bodied herbivores are widely introduced as a way to control weeds (native or exotic) in conservation or restoration projects (reviewed in, for example, Rosenthal *et al*. 2012). Such introductions often amount to *de facto* rewilding projects; e.g. where hardy breeds of livestock replace recently extinct, native mammalian megafauna. However, I am unaware of examples, where a non-native megaherbivore was specifically introduced to provide a novel ecological function that had not previously been present in the ecosystems. Specific lessons from such projects for my proposed use of tortoises on islands are thus difficult to draw, and are beyond the scope of this proposal.

### Island megaherbivores

Megaherbivores are usually defined as mammals weighing more than 1000 kg (Owen-Smith 1988). Their activities have pervasive effects in their ecosystems, often making them keystone species or ecosystem engineers. This is easy enough to envision for hulking beasts like elephants which topple trees, move soil and structure the vegetation (Haynes 2012), literally re-seed their own forest by dispersing large amounts of seeds over great distances (Campos-Arceiz and Blake 2011) and create heterogeneous habitats that can harbour many different smaller species (Pringle 2008; Samways and Grant 2008).

**Figure 1.**
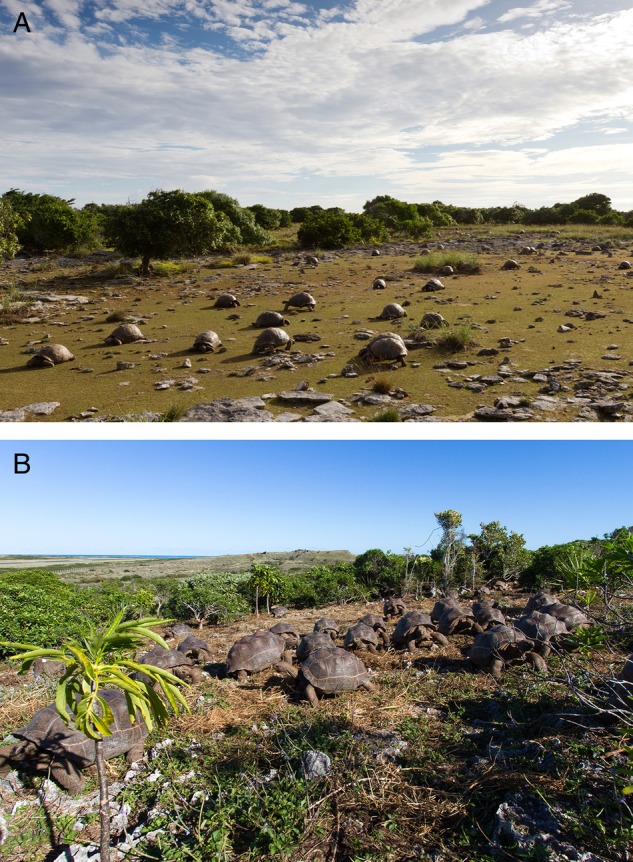
Giant Aldabra tortoises, *Aldabrachelys gigantea*. (A) Grazing tortoises in a high-density region of their native Aldabra Atoll. (B) Newly released herd of subadult tortoises in the rewilding project at the François Leguat Reserve in Rodrigues.

But elephants and their fellow mammalian megaherbivores are never found on isolated oceanic islands. Instead, before human arrival, the largest native vertebrates were typically now-extinct giant species of lizards, flightless birds or tortoises. Especially tortoises were often the largest, or among the largest, vertebrates in many island ecosystems (Hansen *et al*. 2010). However, the ecological effects of large and giant tortoises on islands are equal, in relative scale, to those of elephants in continental ecosystems. Tortoises can thus be thought of as megaherbivores and ecosystem engineers by island standards (Hansen and Galetti 2009). For example, in the Galápagos Islands, giant tortoises move large quantities of seeds (Blake *et al*. 2012), structure the vegetation (Gibbs *et al*. 2010) and create habitats for other species (Froyd *et al*. 2014). Likewise, on Aldabra Atoll, giant tortoises create and maintain large-scale vegetation dynamics (Hnatiuk *et al*. 1976; Merton *et al*. 1976) (Fig. 1A).

### Conservation translocations of island megaherbivores: lessons from tortoise rewilding

In heavily defaunated island ecosystems that are also struggling with invasive plants, rewilding with carefully selected non-native species as functional replacements is increasingly considered a solution, hereby reinstating a herbivory regime that largely benefits the native flora (Hansen 2010; Griffiths *et al*. 2013*a*). Specifically, there is a focus on using extant large and giant tortoises as low-risk, high-impact taxon substitutes (Hansen *et al*. 2010; Burney 2011). So far, tortoise rewilding projects have been initiated on several islands in the Western Indian Ocean (Jones 2008; Hansen *et al*. 2010), and in the Galápagos Islands (Hunter *et al*. 2013). While most of these projects are currently limited in spatial scope to a maximum of a few hundred hectares, there are serious plans to rewild tortoises in much larger areas in the near future, including large areas of up to 15 000 ha in Madagascar (Pedrono *et al*. 2013).

One of the most ambitious examples comes from the isolated island of Rodrigues, 1500 km east of Madagascar. Only slightly more than 300 years ago the French Huguenot François Leguat, one of the island's first human settlers and an avid naturalist, recorded his observations of ‘such plenty of Land-Turtles … that sometimes you see two or three thousand of them in a Flock’ (Leguat 1708). Rodrigues has since lost its endemic giant *Cylindraspis* tortoises, along with the majority of other native animals, most native plant habitat and several plant species (Cheke and Hume 2008). Much of the island today is reduced to secondary grass- and scrubland, heavily overgrazed by cows, sheep and goats (Gade 1985). Recently turning the tide, since 2006 more than 186 000 seedlings from 39 species of native and endemic plants have been planted on 20 ha, forming the François Leguat Reserve, named after the island's first naturalist. Several hundred giant Aldabra tortoises, *Aldabrachelys gigantea*, as well as radiated tortoises, *Astrochelys radiata*, that were initially kept in a smaller enclosed part of the reserve, are now being gradually released into the growing forest (Griffiths *et al*. 2013*b*) (Fig. 1B).

Another example is found on the aforementioned Round Island, Mauritius, where endemic *Cylindraspis* tortoises, extinct on mainland Mauritius since the 1720s, survived to as late as 1844 (Cheke and Bour 2014). Since then, the island's 219 ha were devastated by introduced goats and rabbits. After successful eradications, mostly invasive and introduced plant species gained ground, while especially native grasses continued to decline (North *et al*. 1994; Bullock *et al*. 2002). From 2007 to 2011, a total of 232, mostly juvenile *A. gigantea*, as well as 12 adult *A. radiata*, have been introduced to the island. Feeding observations and analyses of faecal samples revealed that tortoises overwhelmingly eat non-native plants, while ignoring seedlings and saplings of the many naturally regenerating and planted native species (Jones 2008; Griffiths *et al*. 2010, 2013*a*). Until now, despite intensive monitoring, there is no evidence to suggest any major negative impacts on the native biota (Griffiths *et al*. 2011).

The perhaps most controversial application of tortoises as ecological replacements so far, at least in terms of relatedness between extinct and extant taxa, is found in the Makauwahi Cave Reserve on Kaua'i, Hawaii. Here, based on a detailed palaeoecological record and mirroring the efforts in Rodrigues, a forest with native plants is being recreated from scratch (Burney and Burney 2007; Burney 2010). Unlike Rodrigues, though, the native megaherbivore of Kaua'i was not a tortoise, but a giant, flightless duck. However, the unusually broad, powerful beak of the duck had led palaeobiologists to name it the tortoise-jawed moa-nalo, *Chelychelynechen quassus*, suggesting that ‘their ecological role … [was] … probably very closely analogous to tortoises’ (Olson and James 1991). Hence, when faced with increasing problems with invasive plant species in the newly planted forest, the conservationists turned to the idea of using tortoises as low-risk functional replacements. Today, 16 sulcata tortoises, *Centrochelys sulcata*, and two leopard tortoises, *Stigmochelys pardalis*, are being trialled as functional replacements and conservation herbivores in the securely fenced reserve, with promising initial results (Burney *et al*. 2012, 2013). Simple paired choice experiments with invasive and native plants showed that, on average, *S. pardalis* tortoises would consume 55 % of invasive species, but only 18 % of native species offered (Burney *et al*. 2012). Results from even such simple trials with herbivores have been informative in accurately predicting herbivore impacts and changes in plant communities elsewhere (Donlan *et al*. 2002). Encouragingly, this turns out to also be the case in the Kaua'i project. A recent field study showed that the preference pattern also held at the community level, as the top-10 list of the most-consumed plant species by the tortoises featured only invasive plant species (Yamamoto 2014).

### Large and giant tortoises: ideal conservation megaherbivores on islands?

There are two core assumptions underlying my proposed use of non-native large and giant tortoises as megaherbivores to control invasive plant species on islands: (i) they represent easily managed, low-risk conservation translocations, and (ii) the herbivore regime they establish would favour native and endemic plants over invasive ones. Encouragingly, many of the applicability- and risk-assessment issues discussed elsewhere for large and giant tortoises in rewilding projects are directly applicable to island megaherbivore projects (Hansen *et al*. 2008, 2010; Griffiths *et al*. 2010). From these discussions, the most important points in favour of using tortoises as island megaherbivores are:

- Tortoises are easy to breed and rear in captivity, headstarting greatly improves post-translocation establishment
- Natural distribution ranges of available taxa span a great range of suitable habitats and climates (see also Table 1)
- Tortoises are cheap and easy to fence in
- Seasonal use is facilitated by comparative ease of transport and simplicity of husbandry infrastructure required
- The extreme ease of control or removal of tortoises, if needed

**Table 1.** Candidate tortoise-ICM taxa. Lengths are maximum straight carapace lengths in the wild (from same references reported in Hansen *et al*. 2010), IUCN status taken from van Dijk *et al*. (2012). Some large tortoise species might not be very suitable as ICMs, and have not been included here; for example, gopher tortoises, *Gopherus* spp., create burrows that could interfere with restoration goals on degraded islands, and the African sulcata tortoise, *Centrochelys sulcata*, fight if kept at too high densities.

Taxon	Length (cm)	Origin	Native habitat/climate	IUCN status
Giant Aldabra tortoise	105	Aldabra Atoll, Seychelles	Seasonally dry tropics (wild and rewilded)	VU
*Aldabrachelys gigantea*			Wet tropics (rewilded)	
Giant Galápagos tortoise *Chelonoidis nigra* s.l.	75–125	Galápagos Islands, Ecuador	Seasonally dry tropics, humid or dry, lowland or highland, grassland or shrub/forest, depending on taxon	VU to CR, depending on taxon
Yellow-footed tortoise	82	Northern South America	Tropical rain forest	NT
*Chelonoidis denticulata*				
Red-footed tortoise	70	Northern South America	Grassland, dry forest, humid forest	VU
*Chelonoidis carbonaria*				
Asian forest tortoise	60	South-Eastern Asia	Humid tropical forest	EN
*Manouria emys*				
Leopard tortoise *Stigmochelys pardalis*	70	Southern and Eastern Africa	Subtropical dry to temperate, desert to grass- and shrubland, lowland forest, montane forest and grassland	LC
Chaco tortoise	43	Central Argentina	Semi-arid lowland open and scrub forest	VU
*Chelonoidis chilensis*				
Radiated tortoise	40	South Madagascar	Subtropical coastal lowland	CR
*Astrochelys radiata*				
Indian star tortoise *Geochelone elegans*	38	North-western and South-eastern India, Sri Lanka	Dry to humid grassland	VU

Apart from being low-risk, introducing tortoises will likely also be an overall low-cost option, especially for long-term control of invasive plants in sites with high annual costs for chemicals or manual weeding. For example, on Round Island, the high initial cost of translocating tortoises and establishing the population on the island will pay for itself after only 6–7 years, compared with running annual costs of manual weeding (Griffiths *et al*. 2013*a*).

The second core assumption of my proposal is that large and giant tortoises would prefer to eat invasive rather than native plants. As detailed in the previous section, several studies have indeed documented a strong preference for invasive over native plants in tortoise rewilding projects (Burney *et al*. 2012; Griffiths *et al*. 2013*a*; Yamamoto 2014). In general, these preference patterns are most likely explained by invasive fast-growing grasses, herbs and shrubs containing more water or provide higher nutritional value, compared with native plants. It is indeed known that tortoises seek out plants with the highest water content (Peterson 1996). Evidence for a nutritional explanation comes from the Galápagos Islands, an archipelago under increasing threat from invasive plants. Here, where they still occur, endemic giant tortoises actively seek out invasive grasses for their high nutritional value (Blake *et al*. 2015). Lastly, an argument can also be made from a neutral angle: All else being equal, if the plant preferences of generalist large herbivores are ranked by plant abundance, the foraging activity of such herbivores would at any time counteract overabundance by any one or a few plant species.

From a broad biogeographical and evolutionary perspective, certain islands may be especially well-suited for the use of tortoises as megaherbivores because of the source of their flora. For example, native floras of the larger and smaller islands and archipelagos surrounding Madagascar share a relatively high proportion of plant species and lineages with Madagascar (Callmander *et al*. 2011). Madagascar used to harbour several island megaherbivores, including giant tortoises (Goodman and Jungers 2014). It is likely that the flora of these islands, evolutionarily speaking, ‘grew up’ with such megaherbivores, and they may thus retain several of the traits that allowed them to thrive under such herbivore pressures in Madagascar (Bond and Silander 2007; Goodman and Jungers 2014). As a concrete example, the grasslands on many of these islands are dominated by short, tough and dry native grasses and sedges from genera such as *Sclerodactylon* and *Fimbristylis*, while invasive grasses in the region are typically softer, faster-growing species with a comparatively higher water content, such as *Cenchrus ciliaris* and *Dactyloctenium aegypticum*. Island plants with heterophylly (different juvenile and adult leaves) are another good example. The Mascarene Islands are especially rich in heterophyllous plants, with juvenile leaves often being much smaller and brown, or with bright red or purple colour markings (Friedmann and Cadet 1976). This could be a potential anti-herbivory adaptation against being browsed at a vulnerable life stage by the endemic but now-extinct *Cylindraspis* tortoises. Indeed, in choice experiments with *A. gigantea* tortoises, Eskildsen *et al*. (2004) could demonstrate a very clear avoidance of juvenile leaf types by these tortoises even though they are from a different genus native to Aldabra Atoll on the other side of Madagascar.

Following from the above arguments and examples, a general timeline for the empirical application of tortoises as non-native conservation megaherbivores emerges: First, at the most basic level, *ex situ* feeding experiments with candidate tortoise taxa and key invasive and native floral elements can provide rapid quantitative evidence of relative preference patterns. The results can be used to evaluate and select the most appropriate tortoises for the second step: smaller-scale, medium-term *in situ* enclosure experiments. These are essentially inverse versions of commonly performed exclosure experiments to examine impacts of herbivores in ecosystems. The scale of the actual translocation projects could spatially vary from whole-island scenarios (likely on smaller ones only), to residing within smaller, fenced conservation management areas on larger islands. Temporally, the scale could range from seasonal deployment, especially at smaller spatial scales, to the establishment of free-roaming, self-sustaining populations. Empirical data obtained from preliminary smaller-scale deployments can be used to develop detailed individual-based spatially explicit models to help island managers optimize the densities of giant tortoises and predict their impact on the vegetation at larger scales (Hunter and Gibbs 2014).

### Tortoise megaherbivores at the far end of the IUCN conservation translocation spectrum

**Figure 2.**
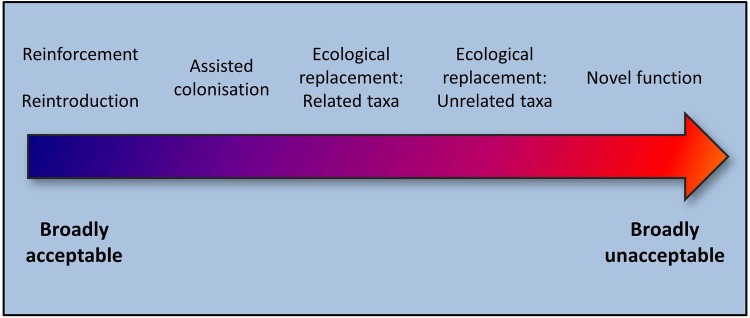
Gradient of acceptability in conservation translocations as defined by the IUCN/SSC (2013) and this study.

A critical issue to keep in mind is that the specifics for every potential project will be idiosyncratic, and that the merits and risks of any proposal need to be evaluated on a case-by-case basis. The IUCN Guidelines for Reintroductions and Other Conservation Translocations (IUCN/SSC 2013) are a useful reference framework to guide decision making. Based on current discussions and opinions in the literature concerning theoretical or real-life applications of conservation translocations, it is perhaps illustrative to think of the relative degrees of contentiousness for the different translocation types as falling along a gradient of acceptability (Fig. 2). Spanning from well-established and broadly acceptable ‘classical’ reintroductions (recreating a locally extinct population using individuals from elsewhere), to assisted colonization (moving species endangered by climate change into more benign climate zones) and to functional replacement (functionally replacing extinct species with related taxa, or, more unacceptable, with functionally similar but unrelated taxa). To some island scientists and practitioners, my suggested use of non-native tortoises to introduce an entirely novel ecological function would no doubt take the place of honour at the far, unacceptable end of the gradient.

This gradient to some extent mirrors the spectrum of decreasing reliance of conservation translocations on historical baselines, suggested by Seddon (2010). This spectrum goes from ‘high’ to ‘low’ reliance as we move from classical reintroductions towards conservation translocations such as assisted colonization and ecological replacements. However, this should not necessarily mean that translocations with a low reliance on historical records are inherently less desireable. As Seddon put it, relying on ‘rigid and often flawed dictates of historical species distribution records’ is not the best way forward for translocation projects, especially in an age of rapid global change. Obviously, my suggestion for tortoise translocations to islands that never had such megaherbivores in the first place stretches Seddon's spectrum a bit further, adding a new ‘none’ at the lower end of it.

Moreover, past ecosystem history is often a baseline that shifts with increasing palaeontological knowledge. The island of Efate in Vanuatu provides a prime example of this. Until recently, even the most avid rewilder had fairly few documented extinct herbivores to consider replacing on Efate, with an omnivore–herbivore scrubfowl, *Megapodius*, being the largest. Any idea of adding giant tortoises to Efate's ecosystem to aid in controlling invasive plants would thus have been out in solid ‘novel function’ territory on the translocation spectrum. But when excavating the earliest layers of human settlements, archaeologists to their surprise found a thick layer of bones of a meiolanid tortoise (an extinct chelonian sister lineage to modern tortoises, turtles and terrapins) with a shell length of more than 1 m (White *et al*. 2010). All of a sudden, translocations of giant tortoises to Efate could now be viewed as less-controversial ecological replacements in rewilding projects (Hansen 2010).

There are already some sites around the world, where conservation translocations of giant tortoises have *de facto* established novel megaherbivore-mediated ecosystem functions. First and foremost in the Seychelles, where at least six islands without historical records of giant tortoises now hold populations of *A. gigantea* (Gerlach *et al*. 2013). One of these is the small island of D'Arros, where current efforts to control invasive plants and restore a coconut plantation to native forest (von Brandis 2012) could likely be helped along significantly by targeted use of the more than 40 adult *A. gigantea* tortoises already living on the island.

### Which tortoises to use, where would they come from and where could they be used?

Extant tortoises are among the most endangered vertebrates worldwide (Böhm *et al*. 2013), but Earth is still home to a number of suitable candidate tortoise taxa, from a broad range of native climates (Table 1). Some of the species listed in Table 1 are specialists that are only found in one particular habitat (e.g. forest tortoises). However, the ecology of many taxa has not yet been studied in detail in the wild, and the distribution ranges of several taxa have shrunk in the last few millennia. The climate and habitat envelopes within which each candidate taxon could be used as island megaherbivores are thus likely broader than Table 1 suggests.

But where would the substantial numbers of tortoises needed come from? The good news is that even for some critically endangered tortoises there are large captive breeding groups that could provide the initial nuclei for sizeable herds. Many of the more common candidate taxa are frequently kept as pets, where individuals that get too large to fit comfortably into a modern household are often shipped off to animal shelters or tortoise sanctuaries. Indeed, such sources were the basis of the small herd of sulcata tortoises used in the Hawaii rewilding trials mentioned earlier (Burney *et al*. 2012). A large potential source of radiated tortoises, *A. radiata*, is found on La Réunion, where they are common household pets, totalling as many as 40 000 island-wide (Boullay 1995).

It is understandable that most people think of tortoises as slow in every way, including reproduction. However, within a relatively short time, many ongoing and planned tortoise rewilding projects in wild or semi-wild conditions would likely also be able to provide significant numbers of tortoises. For example, on Ile aux Aigrettes, since 2003 (2 years after initial release), 11 female and 9 male *A. gigantea* have produced more than 500 hatchlings (Griffiths *et al*. 2012). On the island of Rodrigues, a larger rewilding project with 480 sub-adult and adult *A. gigantea* and 100 adult *A. radiata* released between 2006 and 2009, have produced 568 and 1114 hatchlings, respectively, under natural conditions (Griffiths *et al*. 2013*b*).

In an age of globally ongoing defaunation (Dirzo *et al*. 2014), individuals, organizations and nations that are custodians of breeding populations of large and giant tortoises should thus consider sharing this essentially universal natural heritage with nations or organizations planning restoration projects, where these tortoises could be used as herbivores. Obviously, for an endangered species, *in situ* conservation should still take priority. But even in such cases, translocation or captive breeding could provide animals for island megaherbivore projects elsewhere, affording the species one or several additional retreats from extinction.

Lastly, the huge Aldabra Atoll, Seychelles, located some 400 km north of Madagascar, is home to an optimistic, large-scale tale from the wild. Due to its isolation Aldabra was the last stronghold of Western Indian Ocean giant tortoises. In the early 1800s, when all other species and populations in the region had been harvested to extinction after human settlement, Aldabra still held massive numbers of giant *A. gigantea* tortoises. Accustomed to the taste of their meat, sailors and merchants soon turned up on Aldabra's shores, too, rapidly reducing tortoise numbers on the atoll to a likely low of <1000 tortoises by the late 1800s. At the last minute, in 1874, a group of eminent scientists, including Charles Darwin and Joseph Hooker, wrote a letter urging the protection of the last surviving giant tortoise species in the Indian Ocean, and Aldabra's tortoises were saved. Surprisingly rapidly, the population rebounded to ‘a great many land tortoise all over the place’ by 1929 (Stoddart *et al*. 1979), and all the way back up to likely earlier levels by the late 1960s, when an estimated 120 000–140 000 tortoises once again dominated Aldabra's ecosystem (Cheke and Bour 2014).

The overarching question still begging to be asked is, of course, *where* exactly could tortoises be used as island megaherbivores? Aware of the many idiosyncrasies between islands and island nations with respect to governments, rules and regulations, infrastructure, multiple conservation stakeholders, etc., it is outside the scope of this Point of View to give specific case studies. Instead I have listed a series of biogeographically paired islands or archipelagos with and without a known recent history of native island megaherbivores, respectively (Table 2). They are meant as starting points for a series of thought-experiments that will hopefully stimulate island scientists and practitioners to think outside the box: If arguments that have been put forward for rewilding projects to control invasive plants in the islands *with* a history of native island megaherbivores are deemed to be acceptable and sensible, they should, in principle, be transferable to also succesfully argue the case for using ICMs to control invasive plants in the paired islands *without* a history of native island megaherbivores.

**Table 2.** Examples of biogeographically paired islands or archipelagos with and without a known recent history of native island megaherbivores. See main text for further explanation.

Islands with recent megaherbivores	Islands without recent megaherbivores
Mascarene Islands	Comoros
Madagascar	Îles Éparses
Kaua'i, Hawaii	Leeward Islands, Hawaii
Galápagos Islands, Ecuador	Revillagigedo Islands, Mexico
Pinta, Galápagos Islands	Marchena, Galápagos Islands
Efate, Vanuatu	Tanna, Vanuatu

## Conclusion

As I hope I have been able to explain in the above, my proposal for the introduction of tortoises as island megaherbivores to establish a novel function, is based on ecological functioning, rather than on ecological history and species origin. Ecological and evolutionary history is important for guiding restoration, but in an ever-changing world we need to broaden approaches to not only attempt to restore the past, but also include a strong emphasis on building future resilience, where native plants can prosper under dynamic and flexible biotic regimes. My proposal thus also represents a step in the direction of ‘conciliation biology’ *sensu* Carroll (2011), the eco-evolutionary management of permanently invaded ecosystems.

I am not naively suggesting that the introduction of large and giant tortoises will result in the reduction of all invasive plants on the target islands. But as an adaptive management tool, the novel top-down ecological function provided by tortoises as megaherbivores could help efficiently control invasive species on oceanic islands currently lacking a suitable herbivory regime. The reversibility of trials at even fairly large scales means that nothing is lost from trying, and that much stands to be gained if successful.

To some people's likely immediate ‘but what if they become invasive?’—reaction to the idea of introducing non-native species to islands, I would like to respond with two final tongue-in-cheek remarks. First, I would respectfully suggest that island managers who let large or giant tortoises become invasive on their watch might be doing something wrong. Second, even in a worst-case scenario, not all would be lost, since we could easily adopt control measures employed by Alexandre-Gui Pingré, who was sent to Rodrigues Island in 1761 to observe and record the Transit of Venus. He later reported that, ‘in the three and a half months that I spent on the island, we ate almost nothing else: tortoise soup, fried tortoise, stewed tortoise, tortoise mince sauce, tortoise eggs, tortoise liver […] This meat seemed to me as good on the last day as on the first.’ (Cheke and Bour 2014).

## Sources of Funding

The research was supported by the Institute of Evolutionary Biology and Environmental Studies, University of Zurich.

## Conflict of Interest Statement

None declared.
